# Cholestéatome du méat acoustique externe

**DOI:** 10.11604/pamj.2016.24.269.5977

**Published:** 2016-07-27

**Authors:** Lachkar Azeddine, Ahmed Aabach, Mohamed Chouai, Fahd Elayoubi, Mohamed Rachid Ghailan

**Affiliations:** 1 Service d’Oto-rhino-laryngologie et Chirurgie Cervico-Faciale, CHU Mohammed VI, Oujda, Maroc

**Keywords:** Cholestéatome, méat acoustique externe, paralysie faciale, Cholesteatoma, external auditory meatus, facial paralysis

## Abstract

Le cholestéatome du méat acoustique externe se définit comme une accumulation de kératine en regard d’une érosion osseuse de nature ostéitique. C’est une entité otologique rare ou peut diagnostiquée. Le but de notre travail est d’illustrer sur la base d’un cas un cholestéatome du méat acoustique externe. Il s’agit d’un patient âgé de 65 ans diabétique et hypertendu sous traitement, présentant depuis 3 mois une otalgie droite intense, insomniante, avec hypoacousie, otorrhée purulente minime et paralysie faciale droite grade V. Le diagnostic évoqué était dans un premier temps celui d’otite externe maligne. Il a été mis sous traitement antibiotique sans amélioration. L’examen otologique a trouvé une lésion ulcéro-bourgeonnante de la paroi postérieure du méat acoustique externe droit, une biopsie systématique de la lésion a été pratiquée et a conclu à un cholestéatome. Le patient a bénéficié d’une tympanoplastie en technique ouverte. Le cholestéatome du méat acoustique externe est rare, la symptomatologie clinique n’est pas spécifique, le scanner des rocher est d’un grand apport pour le diagnostic positif montrant un cratère osseux du méat acoustique externe. Le traitement dépend de l’extension des lésions allant des simples soins locaux à une tympanoplastie en technique ouverte. Le cholestéatome du méat acoustique externe peut revêtir plusieurs aspects, et prêter confusion avec d’autres pathologies du méat acoustique externe.

## Abstract (en)

External auditory canal cholesteatoma is defined as an accumulation of keratin leading to osteolytic bony erosion. It is a rare or underdiagnosed otologic entity. Our study aims to illustrate one case of external auditory canal cholesteatoma. Hypertensive diabetic patient, 65 years of age, under therapy suffering from severe right earache, insomnia, hearing loss, small purulent otorrhea and right facial paralysis grade V since 3 months. Initially the diagnosis was malignant external otitis. The patient underwent antibiotic therapy without improvement. Otologic examination showed burgeoning ulcerative lesion of the posterior wall of the right external auditory meatus. Systematic lesion biopsy was performed and confirmed the diagnosis of cholesteatoma. The patient underwent open technique tympanoplasty. External auditory canal cholesteatoma is rare, clinical symptoms are not specific, coronal CT scan makes a major contribution to a positive diagnosis showing bony crater of external auditory meatus. Treatment depends on the extent of the lesions. It can be based on simple local cares or on open technique tympanoplasty. External auditory canal cholesteatoma may have multiple clinical aspects and lend to confusion with other pathologies of the external auditory meatus.

## Introduction

Le cholestéatome du méat acoustique externe se définit comme une accumulation de kératine en regard d'une érosion osseuse de nature ostéitique. C'est une entité otologique rare ou peut diagnostiquée. Le diagnostic de cette affection principalement clinique, a bénéficié des progrès de l'imagerie, permettant de les différencier des autres pathologies inflammatoires et tumorales du méat acoustique externe [1]. Le but de notre travail est d'illustrer sur la base d'un cas un cholestéatome du méat acoustique externe.

## Patient et observation

Il s'agit d'un patient âgé de 65 ans diabétique depuis 25 ans sous antidiabétiques oraux, et hypertendu depuis 20 ans sous traitement, présentant depuis 3 mois une otalgie droite intense, insomniante, irradiant vers la tempe et la région mastoïdienne, avec hypoacousie et otorrhée purulente minime, sans vertige ni acouphène. le diagnostic évoqué était dans un premier temps celui d'otite externe maligne. Il a été mis sous traitement antibiotique pendant 2 mois sans amélioration. Le malade a été adressé dans notre structure pour complément de prise en charge. L'examen otologique a trouvé une lésion ulcéro-bourgeonnante de la paroi postérieure du méat acoustique externe droit avec otorrhée minime, le tympan a été normal. Par ailleurs on a noté une paralysie faciale droite grade VFigure 1. L'audiométrie a objectivé une surdité de transmission modérée. Le scanner des rochers a mis en évidence un épaississement tissulaire de la paroi postérieure du méat acoustique externe avec érosion du tympanal et de la mastoïde en regard Figure 2, Figure 3. Une biopsie systématique de la lésion a été pratiquée et a conclu à un cholestéatome. Le patient a bénéficié d'une tympanoplastie en technique ouverte.

**Figure 1:**
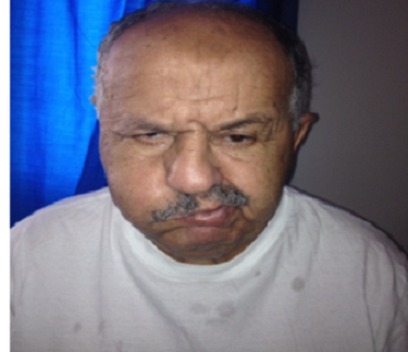
Paralysie faciale droite grade V

**Figure 2:**
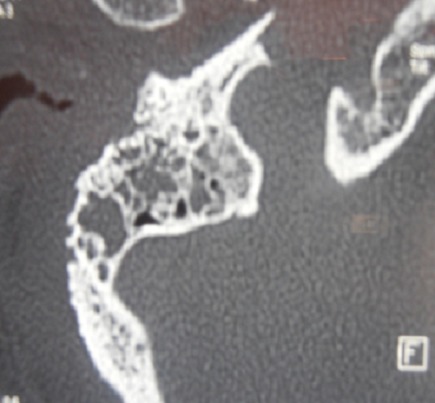
TDM du rocher en coupe axiale: comblement otomastoïdien et lyse de la paroi postérieure du méat

**Figure 3:**
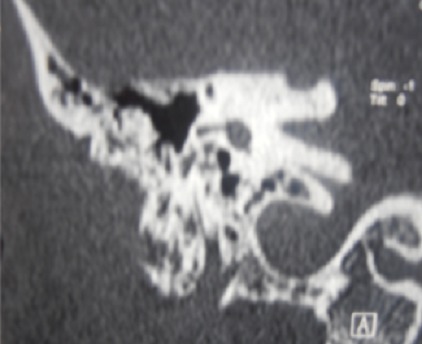
TDM du rocher, coupe frontale: érosion de la mastoïde

## Discussion

Contrairement au cholestéatome de l'oreille moyenne, celui du conduit auditif externe est très rare avec une incidence de 0,1 à 0,5 % de la pathologie otologique. Il est le plus souvent iatrogène. Le cholestéatome primitif est défini par une accumulation kératinique en regard d'une érosion osseuse de nature ostéitique du conduit. Il pose des problèmes de diagnostic positif, de diagnostics différentiels et thérapeutiques. Son étiopathogénie est encore mal élucidée. Plusieurs hypothèses étaient avancées: une périostite localisée, une inflammation chronique du méat acoustique externe, un défaut d'élimination spontanée de l'épithélium desquamé et une déhiscence des sutures tympano-squameuses. Ainsi se dégage la notion d'une atteinte osseuse primitive qui peut être infectieuse ou ischémique et qui engendre la nécrose et la périostite réactionnelle ou l'inverse [2, 3].

L'étude anatomopathologique confirme la présence de périostite limitée à la zone érodée, d'ostéite, de séquestres osseux et de tissu inflammatoire réactionnel. L'accumulation de kératine peut constituer un sac dans une logette osseuse ou auteur d'un séquestre, et progressivement gagner la mastoïde. Cette entité se voit surtout chez le sujet âgé sans différence de sexe, le plus souvent unilatérale. La symptomatologie clinique n'est pas spécifique, faite d'otalgie, d'otorrhée, parfois d'hypoacousie et de paralysie faciale, et parfois de découverte fortuite. L'examen clinique trouve un conduit érodé le plus souvent dans sa paroi inférieure ou postérieure, cette érosion est occupée par des squames et des séquestres. Le tympan peut être normal ou envahi, fonction de l'extension du cholestéatome. L'audiométrie peut être normale ou montrer une légère surdité de transmission [4].

Le scanner est d´un grand apport pour le diagnostic positif montrant un cratère osseux du méat acoustique externe avec intégrité de l´oreille moyenne. Elle permet aussi d'établir un bilan d'extension des lésions à l'oreille moyenne, à la mastoïde, à l'articulation temporo-mandibulaire, à l'oreille interne et au nerf facial [5]. Cependant parfois la biopsie devient indispensable pour apporter le diagnostic. Le cholestéatome du méat acoustique externe doit être différencié des autres pathologies du méat qui peuvent avoir une présentation clinique similaire. Il faut tout d'abord différencier le cholestéatome spontané du cholestéatome secondaire post-traumatique ou iatrogène, un bouchon de cérumen, une ostéite circonscrite du méat, un kyste épidermique, ou encore une lésion néoplasique.

Le traitement dépend de l'extension des lésions. Quand la lésion est limitée, des soins locaux et un débridement suffit. Si la lésion est volumineuse, le recours à une tympanoplastie en technique ouverte avec ou sans plastie du conduit devient obligatoire. Les critères pour la chirurgie incluent des douleurs chroniques, l'échec du traitement médical, les infections récidivantes, le cholestéatome compliqué de paralysie faciale ou de vertige, et l'extension du cholestéatome malgré un traitement médical bien conduit [1].

Le suivi à long terme est nécessaire. Quand une technique ouverte est réalisée, une surveillance par microscope est suffisante. Pour les techniques conservatrices un scanner de contrôle sera réalisé qui servira de référence pour la surveillance.

## Conclusion

Le cholestéatome du méat acoustique externe est une affection rare, pouvant revêtir plusieurs aspects, et prêter confusion avec d'autres pathologies du méat acoustique externe. Son diagnostic est clinique, et son bilan d'extension se base sur le scanner. Le traitement est chirurgical, il dépend du site et de l'étendue des lésions [1, 5].
